# Macrophages Inability to Mediate Adherent-Invasive *E. coli* Replication is Linked to Autophagy in Crohn’s Disease Patients

**DOI:** 10.3390/cells8111394

**Published:** 2019-11-05

**Authors:** Anthony Buisson, Clara Douadi, Lemlih Ouchchane, Marion Goutte, Jean-Pierre Hugot, Anaëlle Dubois, Régine Minet-Quinard, Damien Bouvier, Gilles Bommelaer, Emilie Vazeille, Nicolas Barnich

**Affiliations:** 1 Microbes, Intestine, Inflammation and Host susceptibility (M2iSH), University of Clermont Auvergne/Inserm U1071; USC-INRA 2018, Clermont-Ferrand 63001, France; clara.douadi@uca.fr (C.D.); mgoutte@chu-clermontferrand.fr (M.G.); anaelle.dubois@gmail.com (A.D.); nicolas.barnich@uca.fr (N.B.); 2 Inserm, 3iHP, University Hospital Clermont-Ferrand, Hepato-Gastro Enterology Department, University of Clermont Auvergne, Clermont-Ferrand 63100, France; bommelaer.gilles@orange.fr; 3 CNRS, SIGMA Clermont, Pascal Institute, University of Clermont Auvergne, University Hospital Clermont-Ferrand, Clermont-Ferrand 63000, France; lemlih.ouchchane@uca.fr; 4 UMR843, Inserm, Assistance Publique Hôpitaux de Paris et Université (AP-HP), Paris Diderot, Paris 75013, France; jp.hugot@orange.fr; 5 Biochemistry laboratory, University Hospital Estaing, Clermont-Ferrand 63100, France; rquinard@chu-clermontferrand.fr (R.M.-Q.); dbouvier@chu-clermontferrand.fr (D.B.)

**Keywords:** Crohn’s disease, macrophages, adherent-invasive *E. coli*, *IRGM*, *ULK-1*, autophagy

## Abstract

The macrophages from Crohn’s Disease (CD) patients are defective to control the replication of CD-associated adherent-invasive *E. coli* (AIEC). We aimed to identify the host factors associated with AIEC replication focusing on polymorphisms related to autophagy. Peripheral blood monocyte-derived macrophages (MDM), obtained from 95 CD patient, 30 ulcerative colitis (UC) patients and 15 healthy subjects, were genotyped for several CD-associated polymorphisms. AIEC bacteria survival increased within MDM from CD patients compared to UC (*p* = 0.0019). AIEC bacteria survival increased in patients with CD-associated polymorphism *IRGM* (*p* = 0.05) and reduced in those with CD-associated polymorphisms *XBP-1* (*p* = 0.026) and *ULK-1* (*p* = 0.033). AIEC infection led to an increase of pro-inflammatory cytokines IL-1β (*p* < 0.0001) and TNF-α (*p* < 0.0001) in CD macrophages. ULK-1 expression increased in AIEC-infected MDM from CD patients compared to MDM from UC patients or healthy subjects (*p* = 0.0056) and correlated with AIEC survival (*p* = 0.0013). Moreover, the expression of ULK-1 phosphorylation on Serine 757 decreased following to AIEC infection (*p* < 0.0001). Short-term silencing of *ULK-1* and *IRGM* genes restricted and promote, respectively, AIEC survival within MDM (*p* = 0.0018 and *p* = 0.0291). In conclusion, the macrophage defect to mediate AIEC clearance in CD patients is linked to polymorphisms related to autophagy such as *IRGM* and *ULK-1*.

## 1. Introduction

Crohn’s disease (CD) is a chronic inflammatory bowel disease (IBD) that can lead to altered quality of life and a high level of disability for the patients [1,2]. The etiology of CD remains not fully elucidated. The most admitted theory considers CD as resulting from an abnormal interaction between microbiota and enteric immune system in patients carrying genetic predispositions and influenced by environmental factors. The composition of the microbiota is altered in patients with CD [3]. The role of proteobacteria, and more specifically adherent and invasive *E. coli* (AIEC) has been suggested by several independent studies [4]. AIEC are able to adhere to and to invade epithelial cells lines [5,6]. In vitro studies have demonstrated that CD-associated AIEC are able to survive and replicate within macrophages, leading to increased secretion of tumor necrosis factor alpha (TNF-α) by infected macrophages [7,8,9,10,11,12,13,14]. We recently reported that AIEC bacteria were able to replicate within monocytes-derived macrophages (MDM) from CD patients but not within MDM from ulcerative colitis (UC) patients or healthy controls [14]. This observation suggests that CD MDM are deficient to control intracellular bacteria leading to specific inflammatory response [14]. Elliott and colleagues also reported that MDM retrieved from CD patients were deficient to control several strains of *E. coli* including AIEC strains compared to MDM from healthy controls. The authors supported the hypothesis that macrophage dysfunction was a characteristic feature of CD, rather than the consequence of a specific role of AIEC in stimulating differential macrophage cytokine production in CD [15].

Many genetic variants have been identified as CD susceptibility factors [16]. Some of them could affect directly the function of macrophages. In vitro studies have shown the impact of CD-associated polymorphisms related to autophagy [12,16,17,18,19,20], unfolded protein system [21] and ubiquitin-proteasome system [22] on AIEC survival.

In the present study, we aimed to identify the host factors associated with AIEC survival including genetics parameters and to decipher the mechanisms linking the identified host factors and the defect of macrophages from CD patients to control AIEC infection.

## 2. Materials and Methods

### 2.1. Ethical Considerations

The study was performed in accordance with the Declaration of Helsinki, Good Clinical Practice and applicable regulatory requirements. The study was approved by IRB “*Comité de Protection des Personnes (CPP) Sud-Est 1*”—France (014-33).

### 2.2. Patients

A total of 95 CD patients, 30 UC patients and 15 healthy controls were prospectively and consecutively enrolled between December 2014 and February 2016, at the University Hospital of Clermont-Ferrand, France. Blood samples (50 mL) were drawn from all participants in EDTA tubes regarding MDM separation and in dry tubes regarding genetics. All subjects were genotyped for the main coding mutations (single nucleotide polymorphism = SNP) in *NOD2* [23,24] (rs2066844 (snp8) [Arg702Trp], rs2066845 (snp12) [Gly908Arg] and rs2066847 (snp13) [Leu1007 fsins C]), *ATG16L1* [25] (rs2241880) [T300A], *IRGM* [17,19,26] (rs10065172) [c.313C>T], *ULK1* [20] (rs12303764), *LRRK2* [27,28] (rs11175593), *XBP1* [29] (rs35873774), *CYLD* [30] (rs17314544, rs2302759, rs12324931, rs7205423) and *USP40* [30] (rs12472244, rs4047198, rs838548).

### 2.3. Bacterial Strains

AIEC strain LF82 was isolated from a chronic ileal lesion of a patient with CD [6]. The *E. coli* K-12 C600 strain was used as a non-pathogenic reference.

### 2.4. MDM Isolation and Culture

Monocytes were purified from blood by Ficoll (Eurobio, Coutaboeuf, France) density gradient separation and by negative selection using the EasySep™ Human Monocyte Enrichment Kit (Stem Cell, Grenoble, France). Monocytes were suspended in RPMI 1640 medium (Dutscher, Brumate, France) supplemented with 10% heat-inactivated fetal calf serum (FCS; Dutscher, Brumate, France), 1% l-glutamine (Life Technologies, Carlsbad, CA, USA), and 0.2 μg/mL of recombinant human macrophage colony stimulating factor (rh-M-CSF, Immunotools, Friesoythe, Germany). Cells were seeded into 48-well culture plates at a density of 2.5 × 10^5^ and were incubated at 37 °C in a humidified 5% CO_2_ atmosphere for six days.

### 2.5. MDM Internalization and Survival Assays

MDM were infected at a multiplicity of infection (MOI) of 100. After 10 min of centrifugation at 1000× *g* and a 10 min incubation period at 37 °C with 5% CO_2_, fresh cell culture RPMI 1640 medium, supplemented with 10% heat-inactivated FCS and containing 20 μg/mL of gentamicin, was added for a period of 40 min (1 h post-infection), 6 h or 10 h (6 h or 10 h post-infection). Then, the number of intracellular bacteria was determined as previously described [11,14].

### 2.6. Enzyme-Linked Immunosorbent Assay

At 10 h post-infection, supernatants were collected, centrifuged, and stored at −80 °C. The amounts of IL-1β, IL-6, IL-10 and Tumor Necrosis Factor (TNF)-α released in cell culture supernatants were determined by ELISA (R&D systems, Minneapolis, MN, USA). Cytokine concentrations were assessed according to the manufacturer’s instructions.

### 2.7. Short-Term Silencing of ULK-1 and IRGM Genes

On the fourth day of culture, macrophages were washed with Opti-MEM I Reduced Serum Medium (Invitrogen, Carlsbad, CA, USA) and transfection of siRNA against ULK-1 (Cell signaling technology, Danvers, MA, USA), IRGM (Abcam, Cambridge, UK) or control CTL (Dharmacon, Lafayette, CO, USA) as internal control, at a concentration of 50 nM, was performed using Lipofectamine 2000 (Invitrogen, Carlsbad, CA, USA). At 6 h post-transfection, macrophages were washed with PBS before being suspended in RPMI 1640 medium supplemented with 20% heat-inactivated fetal calf serum and 1% l-glutamine.

### 2.8. Western Blotting

At times of post-infection, monolayers were washed with ice-cold PBS and then scraped in NP40 lysis buffer (25 mmol/L Tris HCl, 150 mmol/L NaCl; 1 mmol/L EDTA; 5 mmol/L EGTA; 1 mmol/L MgCl_2_; 10% Glycérol; 1% NP40; Plus complete protease inhibitor cocktail, Roche; 5 µL/mL PMSF; 10 µL/mL Sodium Orthovanadate; 5 mmol/L NEM, Sigma Aldrich, Saint-Louis, MO, USA). Whole cell extracts were subjected to SDS-PAGE on 12% or 18% acrylamide gels. Proteins were electroblotted onto nitrocellulose membranes (Amersham International, Little Chalfont, UK) and the membranes were immunoblotted for IRGM (rabbit, 1:1000, Abcam, Cambridge, UK), SQSTM1/p62 (mouse, 1:1000, Santa Cruz Biotechnology, Dallas, TX, USA), ULK-1, p-ULK-1 Ser757, LC3A/B and GAPDH as internal control (all rabbit, 1:1000, Cell signaling technology, Danvers, MA, USA). Immuno-reactants were detected using horseradish peroxidase-conjugated anti-rabbit or anti-mouse immunoglobulin G antibody, ECL reagents (Abcam, Cambridge, UK), through a ChemiDoc camera (Bio Rad, Hercules, CA, USA). Image J software was used to estimate protein quantity. Results were expressed as protein amount relative to GAPDH and to uninfected or transfected against siCTL MDM.

### 2.9. Sample Size Calculation

The sample size has been calculated from the preliminary data [14] and regarding the primary endpoint, i.e., to identify the genetic factors associated with AIEC survival, with a two-sided type I error of 5% and a power of at least 80%. According to our preliminary results founding a mean replication of 3.25 ± 5.54 (SD), we estimated that 95 patients with CD would enable showing an effect size from 3 to 7 depending on the prevalence of polymorphisms, which seems to us scientifically relevant. In addition, we planned to also include patients with UC and healthy controls in order to test for possible mutation effect and also replicate results from a previous study [14]. Then, we also based our sample size and power calculations on these analyses, with a particular emphasis on the latter objective, as the first one was more exploratory. We scheduled to enroll 145 subjects with 70%, 20% and 10% of CD patients, UC patients and healthy controls, respectively. Considering disease effect assessed through a one-way ANOVA, power is estimated at 0.8 or over for marginal effect (power = 0.8), comparison of CD patients versus the other groups (power = 0.812) and comparison of CD versus UC patients (0.811).

### 2.10. Data Collection and Statistical Analysis

Study data were collected and managed using REDCap electronic data capture tools hosted at the University Hospital of Clermont-Ferrand. REDCap (Research Electronic Data Capture) is a secure web-based application designed to support data capture for research studies, providing: 1) an intuitive interface for validated data entry; 2) audit trails for tracking data manipulation and export procedures; 3) automated export procedures for seamless data downloads to common statistical packages; and 4) procedures for importing data from external sources.

The statistical analyses were performed on SAS (SAS v9.4, Cary, NC, USA). All statistical tests were conducted with a two-sided type I error set at 0.05. Continuous variables were described as mean and standard deviation (SD) or median and interquartile range when appropriate, according to statistical distribution (assumption of normality studied using Shapiro-Wilk test). We performed Spearman correlation coefficient tests to seek for link between continuous variables. Other univariate analyses were performed using Student *t*-test or Mann-Whitney test when assumptions of *t*-test were not met (normality, homoscedasticity assessed by the Fisher-Snedecor test) for continuous variables, and chi-square or Fisher’s exact tests for categorical data. Comparisons of continuous variables between more than two groups were performed through one-way ANOVA (or Kruskall Wallis’ test when appropriate), eventually completed by multiple comparisons procedure using Tukey grouping. The univariate analyses were focused on patients with CD. It means that we assessed the relationship between each parameter (patients’ demographics, markers of disease activity (see Table 1) and genetic factors) and AIEC internalization or survival among the 95 CD patients. For example about IRGM, we compared AIEC survival in patients carrying the risk variant (*n* = 27 patients) versus those who did not (*n* = 68). Multivariate analyses were carried out in order to account for possible confounding factors. The studied factors are listed in Table 1. However, we also evaluated whether the impact of the identified factors was restricted to one subgroup (CD, UC or HS) in performing interaction tests.

## 3. Results

### 3.1. Clinical and Genetics Characteristics of the Cohort

The clinical characteristics of the included patients and healthy subjects are detailed in Table 1. The prevalence of the different investigated CD-associated polymorphisms is reported in Table 2.

### 3.2. The Internalization of AIEC Bacteria Was Not Influenced by the Origin of the Monocytes-Derived Macrophages but Was Lower in Patients Carrying the CD-Associated Polymorphism CYLD

**Figure 1:**
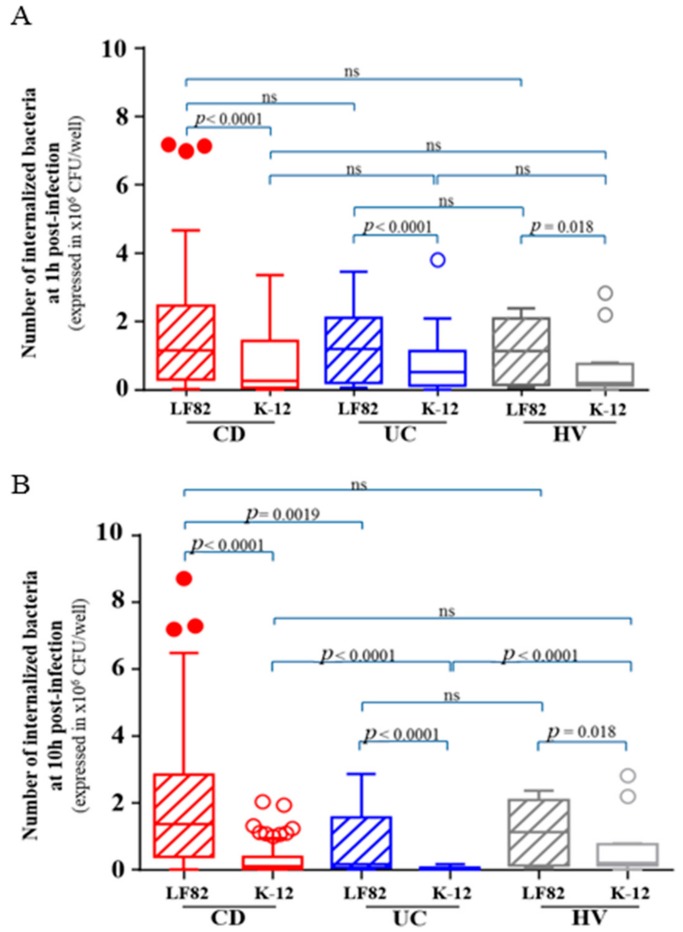
Number of internalized bacteria within macrophages at 1 h post-infection (**A**) and 10 h post-infection (**B**) in 95 CD patients, 30 ulcerative colitis patients and 15 healthy subjects. Statistical analysis was performed using Kruskall Wallis’ test and multiple comparisons and figure are performed using Tukey grouping. ns = not statistically significant.

The internalization of AIEC reference strain LF82 and non-pathogenic *E. coli* K-12 C600 strain by macrophages, defined as the number of intracellular bacteria within macrophages at 1 h post-infection, was assessed in peripheral blood MDM obtained from 95 CD patients, 30 UC patients and 15 healthy subjects. A higher number of intracellular AIEC LF82 bacteria were internalized within MDM, regardless of their origin, compared to non-pathogenic *E. coli* K-12 C600 bacteria (*p* < 0.0001) (Figure 1A). Of note, the number of internalized AIEC bacteria at 1 h post-infection was not different according to MDM origin (CD, UC or healthy controls) (Figure 1A). We investigated the factors influencing the internalization of AIEC bacteria. In univariate analysis, we did not find any relationship between AIEC internalization and clinical parameters or markers of disease activity (CDAI/SCCAI, CRP or calprotectin) while the risk variant *CYLD* rs2302759 (*p* = 0.015) was associated with decreased number of internalized AIEC bacteria within macrophages. The CD-associated polymorphism *XBP-1* and *ULK-1* exhibited also a trend to be associated with a decreased AIEC bacteria internalization (*p* = 0.074 and *p* = 0.10, respectively).

In multivariable analysis, only *CYLD* rs2302759 risk variant was associated with a reduced number of internalized AIEC bacteria at 1 h post-infection within MDM (*p* = 0.017). In our cohort, the impact of this mutation was not significantly specific of MDM from CD patients (*p* 0.76). Accordingly, these data show that the internalization of AIEC bacteria was higher than non-pathogenic *E. coli* strains and was not influenced by the origin of the MDM, but was lower in patients harbouring the CD-associated polymorphism *CYLD.*

### 3.3. AIEC Bacteria Survival Is Increased within MDM from CD Patients and Was Impacted by the CD-Associated Polymorphisms IRGM, XBP-1 and ULK-1

The survival of AIEC reference strain LF82 and non-pathogenic *E. coli* K-12 C600 strain within macrophages, defined as the number of intracellular bacteria at 10 h post-infection was assessed in peripheral blood MDM obtained from 95 CD patients, 30 UC patients and 15 healthy subjects. We observed a higher number of intracellular AIEC LF82 bacteria within MDM, regardless of their origin, compared with non-pathogenic *E. coli* K-12 C600 bacteria (*p* < 0.0001) (Figure 1B). The AIEC bacteria survival was higher within MDM from CD patients compared to those from UC patients (*p* = 0.0019) (Figure 1B). We investigated the factors influencing the survival of AIEC bacteria within MDM. In univariate analysis, we did not find any relationship between AIEC replication and clinical parameters markers of disease activity (CDAI/SCCAI, CRP or calprotectin). The presence of the risk variants for *XBP-1* (*p* = 0.049), *ULK-1* (*p* = 0.034) and *CYLD* rs2302759 (*p* = 0.035) was associated with decreased number of AIEC bacteria survival within CD macrophages while the risk variant *IRGM* was associated with increased AIEC bacteria survival (*p* = 0.05).

**Figure 2:**
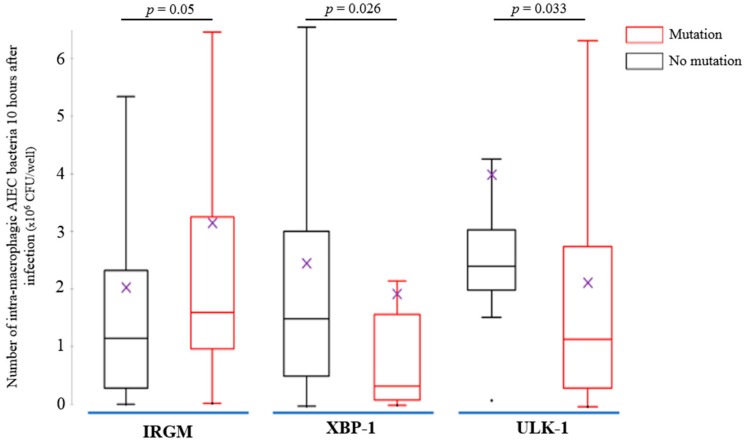
Results of the multivariate analysis of adherent and invasive *E. coli* survival within monocytes-derived macrophages from 95 Crohn’s disease (CD) patients according to the presence of the CD-associated polymorphisms *IRGM* (*n* = 27 patients, 28.4%), *XBP1* (*n* = 13 patients, 13.7%) and *ULK1* (*n* = 82 patients, 86.3%). The comparisons were performed using the Mann-Whitney test and the figure was presented with Tukey grouping. (Means are represented as purple crosses and median as horizontal lines; for IRGM, “no mutation” means patients who are not carrying CD-associated polymorphism IRGM; for XBP-1, “no mutation” means patients who are not carrying CD-associated polymorphism XBP-1; for ULK-1, “no mutation” means patients who are not carrying CD-associated polymorphism ULK-1).

In multivariable analyses, polymorphisms at risk of *XBP-1* (*p* = 0.026) and *ULK-1* (*p* = 0.033) were independently associated with decreased survival of AIEC bacteria within CD MDM (Figure 2). In contrast, MDM from CD patients (*p* < 0.0001) and presence of CD-associated *IRGM* polymorphism (*p* = 0.05) were associated with higher survival of AIEC bacteria (Figure 2). In addition, we did not observe any significant interaction between these three mutations (*ULK-1*, *p* = 0.38; *XBP-1*, *p* = 0.62; *IRGM*, *p* = 0.90) and the origin of the MDM (CD, UC or healthy subjects) regarding the impact on AIEC bacteria survival, meaning that the impact of these polymorphisms are not significantly different between patients with CD, UC and healthy controls.

### 3.4. AIEC Bacteria Induce an Excessive and Disordered Inflammatory Response by Macrophages

**Figure 3:**
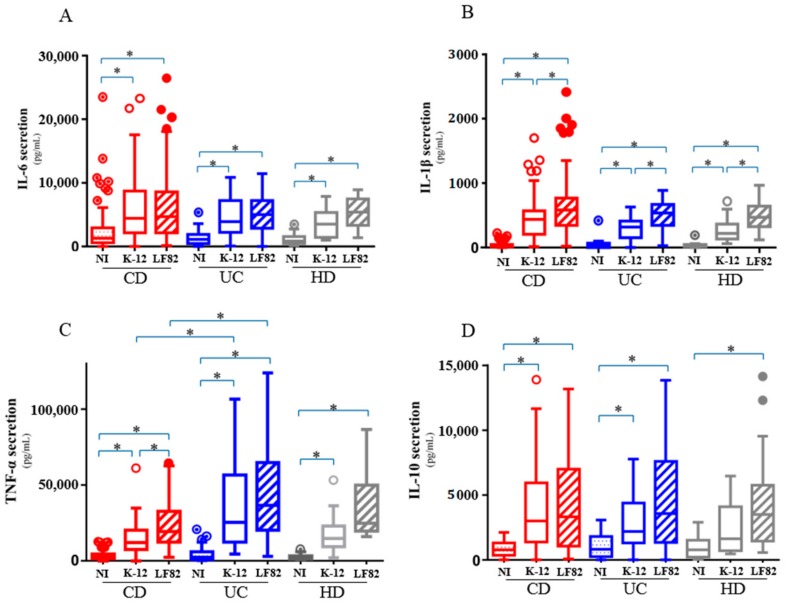
IL-6 (**A**), IL-1β (**B**), TNF-α (**C**) and IL-10 (**D**) cytokines level released in the supernatant of monocytes-derived macrophages from 95 Crohn’s disease patients, 30 ulcerative colitis patients and 15 healthy subjects after 10 h of bacterial infection. (mean ± s.e.m., * *p* < 0.05).

We quantified and compared cytokine secretion by macrophages between the three groups (CD, UC and controls) at the basal state, following AIEC and non-pathogenic K-12 infection. The level of the pro-inflammatory cytokines TNF-α, IL-1β and IL-6 and the anti-inflammatory cytokine IL-10 was quantified at 10 h post-infection in the supernatants of infected MDM. AIEC infection led to significant increased release of pro-inflammatory cytokines IL-1β (Figure 3A) and TNF-α (Figure 3B) compared to *E. coli* strain K-12 infection or uninfected MDM regardless of the MDM provenance (*p* < 0.0001 for both). TNF-α secretion from uninfected or AIEC-infected MDM increased in patients with active CD (CRP value > 5 g/L or faecal calprotectin > 250 µg/g) (*p* = 0.026). In contrast, bacterial infection, irrespective of the type of bacteria i.e., AIEC or *E. coli* strain K-12, induced an increased release of IL-6 in the supernatants of MDM regardless of their origin (*p* < 0.0001 for each of them) (Figure 3C). The level of IL-6 was higher in the CD patients with abnormal CRP level (CRP > 5 g/L) (*p* = 0.008). The level of IL-10 in the MDM supernatants regardless of their origin (CD, UC or healthy subjects) was higher after bacterial infection with no difference between AIEC and *E. coli* strain K-12 (*p* < 0.0001, *p* < 0.0001 and *p* = 0.0003, respectively) (Figure 3D). We looked at the potential relationship between clinical parameters (see Table 1), current medications or genetic parameters, and the level of cytokines. We did not find any other significant correlation than those reported above.

We investigated the correlation between AIEC survival and cytokines production. In MDM from CD patients, a positive correlation between AIEC survival and the level of IL-1β secretion (ρ = 0.54; *p* < 0.0001), IL-6 (ρ = 0.29; *p* = 0.006), IL-10 (ρ = 0.46; *p* < 0.0001) and TNF-α (ρ = 0.31; *p* = 0.003) was observed. Of interest, such correlations were not observed between *E. coli* strain K-12 survival and cytokines production (Table 3). Together, these data suggest that AIEC infection could favor an excessive and unbalanced inflammatory response by macrophages.

### 3.5. ULK-1 Protein Activity and AIEC Bacteria Survival Are Dependent of Each Other in MDM from CD Patients

**Figure 4:**
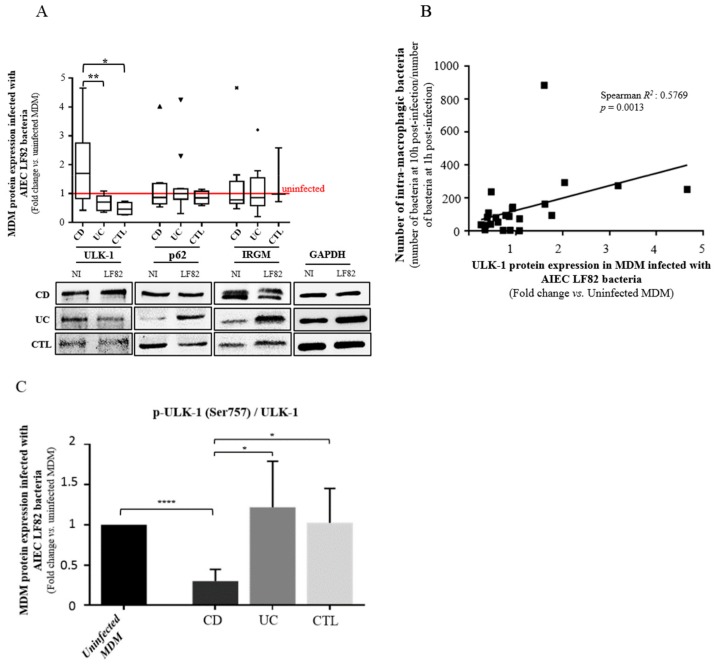
Influence of autophagy-related ULK-1 protein expression and activity on bacterial clearance within MDM from CD patients. (**A**) Western blot and quantification of ULK-1, p62 and IRGM proteins levels in MDM from CD patients (*n* = 10), UC patients (*n* = 12) and healthy volunteers (*n* = 4 for ULK-1 and P62 protein levels and *n* = 3 for IRGM protein level), infected during 10 h or not with AIEC LF82 bacteria. Results are quantified relative to GAPDH. (**B**) Correlation between the number of intra-macrophagic bacteria reflecting the bacteria survival and ULK-1 protein level in MDM infected with AIEC LF82 (*n* = 26). (**C**) Western Blot quantification of p-ULK-1 Ser757 protein level in MDM from CD patients (*n* = 13), UC patients (*n* = 14) and healthy volunteers (CTL) (*n* = 14), infected during 10 h or not with AIEC LF82 bacteria. All the subjects included in this analysis are from the clinical protocol. Results are quantified relative to total ULK-1. (mean ± s.e.m., * *p* < 0.05, ** *p* < 0.01, **** *p* < 0.0001).

Autophagy plays a key role in managing AIEC infection [12,31]. After showing that the CD-associated polymorphisms *IRGM* and *ULK-1* impacted AIEC survival in MDM from CD patients, we decided to confirm these data in vitro with samples of subjects remaining from the clinical protocol. We measured the level of IRGM and ULK-1 proteins as well as the level of p62, which is a protein used as a marker of autophagic flux, in AIEC LF82-infected MDM from CD patients, UC patients and healthy subjects (Figure 4A). Contrary to IRGM and p62, the level of ULK-1 protein was significantly increased within AIEC LF82-infected MDM from CD patients compared to MDM from UC patients or healthy subjects (approximately two-fold higher, *p* < 0.05) (Figure 4A). The level of ULK-1 protein was significantly correlated with the number of AIEC LF82 bacteria at 10 h post-infection within AIEC LF82-infected MDM from CD patients (ρ = 0.37; *p* = 0.033) (Figure 4B). As the level of total ULK-1 protein does not mean there are changes in its activity, we assessed the effect of AIEC on ULK-1 focusing on serine 757 phosphorylation which regulates negatively the autophagy process. We observed a significant decrease of the ratio p-ULK-1 on Ser757/total ULK-1, within macrophages from CD patients following AIEC infection compared to uninfected macrophages (*p* < 0.0001), UC patients (*p* = 0.0212) and healthy volunteers (*p* = 0.0186), highlighting the induction of autophagy in MDM from CD patients (Figure 4C). Also, as a marker of autophagy process, we measured LC3A/B levels in MDM from CD, UC and CTL by western blot. LC3A/B–II expression tends to increase following AIEC LF82 infection compared to uninfected MDM, regardless of the origin, suggesting the induction of autophagy (Figure S1).

### 3.6. The Internalization of AIEC Bacteria is Influenced by the Autophagic Process and Especially the Expression of ULK-1 in MDM

**Figure 5:**
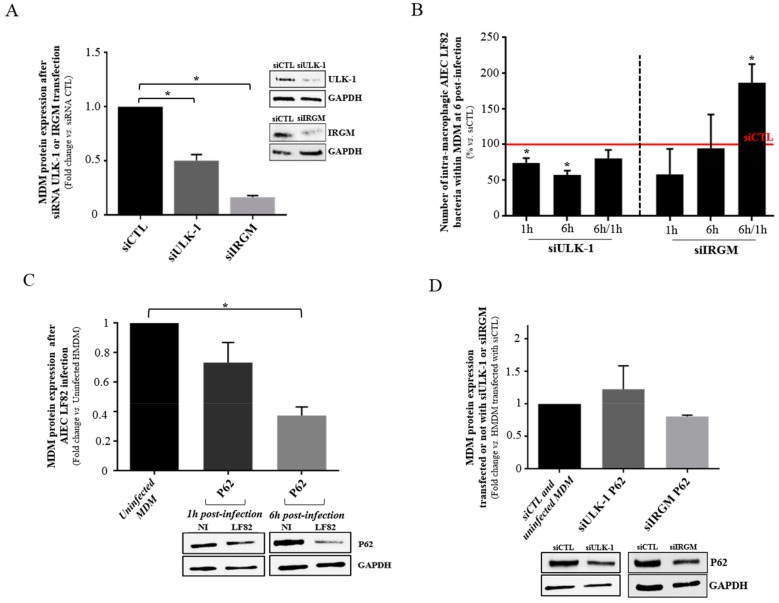
Influence of autophagy-related ULK-1 and IRGM proteins expression on bacterial clearance within MDM from blood donors. (**A**) MDM were transfected with either control siRNA, ULK-1 or IRGM siRNA. ULK-1 and IRGM proteins level was analyzed by western blotting. (**B**) Number of intra-macrophagic AIEC LF82 bacteria at 6 h post-infection within MDM after control, ULK-1 or IRGM siRNA transfection. (**C**) Western blot and quantification of P62 level in MDM infected or not by AIEC LF82 at 1 h and 6 h post-infection. (**D**) Western blot and quantification of P62 level in MDM, after siCTL, siULK-1 or siIRGM transfection after AIEC LF82 infection or not at 6 h post-infection. All MDM were infected by AIEC LF82 at MOI 100. For the western blots, quantification is expressed as amount relative to GAPDH and to uninfected MDM or control siRNA. Data represent results from at least three independent experiments. (mean ± s.e.m., * *p* < 0.05).

To validate the hypothesis of the potential impact of ULK-1 on AIEC clearance within macrophages and to compare it to another common autophagy protein IRGM, a short-term silencing of *ULK-1* and *IRGM* was performed on MDM from blood donors (Figure 5A). We observed a reduced survival ability of AIEC LF82 at 1 h and 6 h post-infection within MDM transfected with siRNA targeting *ULK-1* compared to those transfected with scramble siRNA (*p* = 0.0157 and *p* = 0.0018, respectively) (Figure 5B). By contrast, after siRNA *IRGM* silencing, the replication ability (6 h/1 h ratio) of AIEC LF82 within MDM increased compared to siRNA control (*p* = 0.0291). Besides, we aimed to assess the involvement of autophagy on the clearance of AIEC bacteria by the MDM from blood donors. We analyzed the impact of AIEC LF82 infection on p62 protein accumulation within MDM. We observed a significant decrease of p62 protein expression at 6 h post-infection within AIEC LF82-infected MDM compared to those non-infected (Figure 5C), highlighting the induction of autophagy. As expected, the down-regulation of ULK-1 and IRGM by siRNA prevented autophagy initiation as shown by the absence of p62 decrease at 6 h post-infection (Figure 5D), highlighting an impact of ULK-1 and IRGM on the autophagy process in AIEC-LF82 infected macrophages. In conclusion, these results suggest that AIEC could take advantage of the autophagy machinery at its early step, as ULK-1 is well described as an autophagy starter [32,33], contrary to IRGM.

## 4. Discussion

In this study, we revisited the theory of “dyspeptic macrophages” to partially explain the genesis of CD, in focusing on the ability of these macrophages to handle CD-associated AIEC bacteria. We confirmed that macrophages from CD patients present a specific defect to control AIEC replication, and this is impacted by several CD-associated polymorphisms linked to autophagy. Besides, we showed that AIEC infection led to a loss of balance of cytokine secretion by macrophages.

Conflicting results have been reported regarding the inflammatory response of *E. coli*-infected monocytes/macrophages from CD patients [14,15,34,35,36,37,38,39]. In the present study, we observed an excessive inflammatory response to AIEC infection compared to non-pathogenic *E. coli* exhibiting higher release of TNF-α and IL-1β and similar level of IL-6 and IL-10. We observed that the level of TNF-α was associated with disease activity assessed by objective marker of intestinal inflammation (combination of faecal calprotectin and CRP level) but not clinical activity (CDAI) which is now widely admitted as a suboptimal way to evaluate disease activity [40]. The cytokines production in response to AIEC infection was similar across the three groups (CD, UC and controls) except for TNF-α release between UC and CD patients. The level of cytokines was correlated with the number of surviving bacteria. Our data are in line with those from Schwarzmaier et al. reporting that similar levels of cytokines released, including TNF-α and IL-6, were not specific of macrophages origin, if we only consider CD and healthy subject, but may be related to the degree of intestinal inflammation [41]. In our study, the macrophages from UC patients seem to be more reactive in terms of TNF-α secretion following infection compare to CD patients. However, the strong heterogeneity of levels of TNF-α from UC MDM makes the interpretation of this significance difficult and would need to be investigated in other dedicated studies. In addition, Zorzi et al. reported that the secretion of pro-inflammatory cytokines may possibly vary in CD, in a temporary manner [42]. In our study, we identified different macrophages between CD and UC patients in relationship with the AIEC replication. AIEC replication was correlated with the anti-inflammatory cytokine IL-10 in patients with CD suggesting that AIEC bacteria are able to adapt the macrophages environment to proliferate and promote its own colonization. In contrast, AIEC replication was correlated with pro-inflammatory cytokines in patients with UC (TNF-α and IL-1β).

The analysis of bacterial internalization at 1 h post-infection by MDM revealed that cells obtained from CD patients were not more permissive to bacteria including AIEC and non-pathogenic *E. coli* than MDM obtained from UC patients or healthy controls. Accordingly, a functional phagocytosis of CD-associated monocytes/macrophages have been previously shown to be necessary to fight other pathogens such as *Staphylococcus aureus* and *Candida albicans* [30,43]. Schwarzmaier et al. reported that phagocytosis of non-pathogenic *E. coli* was not impaired in peripheral blood monocytes of patients with inactive CD [41]. Two recent works also supported similar phagocytic activity in MDM from CD patients compared to those from healthy controls [15,39]. In contrast, it was observed in the 1990s, in 20 Japanese patients with CD, using automated laser flow cytometry, a decreased phagocytic activity of monocytes from CD patients especially in case of active disease [44]. Although all of these studies are not fully comparable because of different inclusion criteria and clinical activity, our data advocate for similar MDM properties in handling AIEC bacteria regardless of their origin. In addition, AIEC demonstrated higher ability to penetrate into macrophages compared to non-pathogenic *E. coli* K-12 bacteria, regardless of the groups (CD, UC or healthy subjects). AIEC bacteria may express specific factors that could interact with some receptors expressed by macrophages, in the same way as the interaction between the CEACAM6 receptor and the type 1- pili expressed by AIEC [45] or between the N-glycosylated CHI3L1 and the chitin-binding domain of chiA [46,47] on intestinal epithelial cells. Additional dedicated works could be of great value to better understand what factors could favor AIEC bacteria entrance within macrophages. Our data also highlighted the potential role of the ubiquitin-proteasome system in the AIEC internalization, as the patients presenting the CD-associated polymorphism *CYLD* had a diminished AIEC internalization. It suggests that the AIEC bacteria could take advantage of the ubiquitin-proteasome system to enter within macrophages. However, our results should be taken with caution and should be confirmed in a larger cohort as only two patients carried the risk variant for *CYLD* rs2302759.

It has been well established that CD-associated *E. coli* are able to survive and replicate within murine J774-A1 macrophages, human THP-1 macrophages [12], and human MDM obtained from fresh human blood of healthy controls [7,8,9,10,11,13]. In vitro analysis of the intracellular traffic of bacteria-containing vacuoles in macrophages has revealed that AIEC persist in vacuoles with phagolysosomal traits [7]. In a preliminary study, we suggested that AIEC bacteria were able to survive within MDM from CD patients but not within MDM from UC and healthy controls [14]. In the present study, we confirmed our previous findings. Contrary to a non-pathogenic *E. coli* strain, AIEC bacteria were able to resist to MDM killing, regardless of the cells’ origin. In addition, intracellular replication of AIEC bacteria was only observed in MDM from CD patients. This result points to defects of MDM from CD patients in the ability to restrict the number of intracellular AIEC bacteria, in a large cohort of CD patients. Many years ago, historical works supported the hypothesis of dyspeptic macrophages [43], i.e., the disability to degrade phagocytosed materials with impaired bacterial clearance [35], to partly explain the genesis of CD [43]. More recently, while Elliott and colleagues observed also that MDM from CD patients failed to control AIEC intracellular replication compared to those from healthy controls [15], a small sample size study including only 10 CD patients reported conflicting results [39]. As the deficiency to control AIEC is specific of CD macrophages, we investigated the host factors associated with AIEC survival in CD. We observed in multivariable analysis that the presence of the CD-associated polymorphisms *XBP-1* and *ULK-1* were independently associated with decreased AIEC survival. It could be partly explained by the fact that *XBP-1* mutation lead to a dysfunction of the unfolded protein system which could induce an accumulation of intracellular unfolded protein and then to an increased cell death [29]. In the same way, as ULK-1 is warranted to initiate autophagy, the presence of the CD-associated polymorphism could reduce the internalization of AIEC bacteria [20].

**Figure 6:**
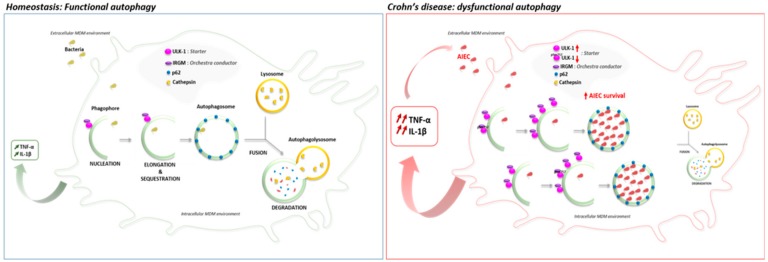
Proposed model of autophagy and cytokines secretion’s role in macrophages infected by AIEC bacteria from Crohn’s Disease patients. In homeostasis state, the internalized bacteria are in part addressed to the functional autophagic process to be degraded by the cathepsins. However, in macrophages from Crohn’s disease patients, the ULK-1 activity increase may lead to an exacerbated dysfunctional autophagy in favor to the AIEC internalization and survival into the autophagosome. In response to AIEC infection, the deficient macrophages secrete an excessive inflammatory cytokine such as, TNF-α and IL-1β, that then benefit to the internalization of AIEC, triggering a detrimental loop of chronic inflammation.

In this line, we aimed to determine the impact of CD-associated polymorphism *ULK-1* at the protein scale. We down-regulated ULK-1 and IRGM expression in MDM from healthy subjects and measured ULK-1 expression in AIEC-infected MDM from CD patients. In the present study, we showed, for the first time that the down-regulation of ULK-1 limits the AIEC survival, contrary to IRGM down-regulation, and prevent p62 degradation, suggesting an impaired autophagy onset [48], in MDM from healthy subjects. Indeed, in contrast, we showed a functional autophagy with an effective p62 degradation in MDM from healthy subjects. It is well established that AIEC survive and replicate into mature phagolysosome in macrophages without inducing cell death, promoting a large secretion of TNF-α [11]. In our context, *ULK-1* polymorphism may cause a specific impaired autophagy onset that could limit the AIEC internalization and consequently, survival into macrophages. Furthermore, we observed that ULK-1 expression was higher in AIEC-infected MDM from CD patients than in infected MDM from UC patients or healthy subjects and positively correlated with the number of internalized AIEC. As ULK-1 activity is dependent on phosphorylation, we showed a significant decrease of the ratio p-ULK-1 on Serine 757/total ULK-1 within macrophages from CD patients following AIEC infection compared to uninfected macrophages, UC patients and healthy volunteers. The phosphorylation of Serine 757 on ULK-1 protein regulates negatively the autophagy process in blocking the ULK-1-AMPK interaction [49]. We hypothesize that in macrophages from CD patients, AIEC bacteria could take advantage of an increase ULK-1 activity, triggering an exacerbate autophagy onset in using a replicative niche within phagolysosomes leading to unbalanced inflammation (Figure 6). In the article by Lapaquette et al., knock-down of ATG16L1 and IRGM led to a defect of autophagy process and an increase of AIEC survival. Although ULK-1, ATG16L1 and IRGM are all implicated in autophagy, they act at different moments as they do not have the same role. ULK-1 is essential for the initiation process while ATG16L1 is indispensable for the elongation process. IRGM orchestrates antimicrobial autophagic responses. In summary, while AIEC bacteria need a functional onset of autophagy to be able to survive and replicate within phagolysosomes, dysregulated autophagy is required to promote AIEC replication. It could be surprising as this *ULK-1* polymorphism has been shown to be associated with higher risk of CD (64% vs. 57%; *p* = 0.002) [20]. However, one third of patients with CD are infected by AIEC bacteria and we hypothesize that the patients carrying the CD-associated *ULK-1* polymorphism may be at decreased risk to be infected by AIEC bacteria. This theory should be confirmed by further investigations.

In contrast, we also showed an increased AIEC survival for patients carrying the CD-associated polymorphism *IRGM*. To our knowledge, the present study is the largest cohort published so far confirming that MDM from CD patients exhibit a defect to control AIEC survival, and the first identifying the CD-associated polymorphism *IRGM* as an associated factor to AIEC survival within MDM from CD patients. Two previous studies failed to show any genetics association because of the small sample size ranging from 10 to 14 CD patients [15,39]. In addition, it has been previously demonstrated that IRGM c.313C>T polymorphism alters the negative regulation induced by microRNA 196 on IRGM, leading to IRGM overexpression, autophagy dysregulation and increase of AIEC survival [12,17,31]. While our results on *ULK1*, which is the starter of autophagy, indicated that AIEC bacteria could take advantage from an exacerbated autophagy onset, a functional autophagy seems to be detrimental for AIEC bacteria. Then, the inactivation of *IRGM*, which is the real orchestra conductor of autophagic machinery [26], favored AIEC replication as confirmed by in vitro assays in intestinal epithelial and human THP-1 macrophages cell lines [14,31].

Several strengths have to be underlined in our study. It is the first and the largest study published so far, confirming the macrophages defect to control AIEC survival and identifying the association with the CD-associated polymorphism *IRGM*. In addition, multivariable analyses were used to investigate the potential role of the patients’ characteristics including genetic factors and the impact of medication. In conclusion, we confirmed, in a large cohort of patients that macrophages from CD patients are deficient to control AIEC survival and identified a role of several CD-associated polymorphisms related to autophagy (*ULK-1*, *IRGM*). We reported a specific inflammatory response after AIEC infection depending on intestinal inflammation. These findings lead to investigate the host-pathogen interaction as a potential therapeutic target in the future.

## Figures and Tables

**Table 1:** Characteristics of Crohn’s disease patients, ulcerative colitis patients and healthy subjects included in the study.

	CD Patients	UC Patients	Healthy Subjects
Age, mean (±SD)	41.3 ± 13.9	43.1 ± 14.6	33.6 ± 10.8
Disease duration at inclusion, mean (±SD)	11.1 ± 9.8	9.9 ± 9.1	-
Body Mass Index, mean (±SD)	24.6 ± 5.5	24.4 ± 4.6	22.5 ± 2.5
Male gender, n (%)	32 (33.7)	17 (56.7)	5 (33.3)
Tobacco use, n (%)			
Non-smokers, n (%)	61 (74.2)	9 (30.0)	11 (73.3)
Former smokers, n (%)	6 (6.3)	18 (60.0)	1 (6.7)
Active smokers, n (%)	28 (29.5)	3 (10.0)	3 (20.0)
Previous intestinal resection, n (%)	30 (31.6)	0 (0.0)	0 (0.0)
Previous appendectomy, n (%)	35 (36.8)	4 (13.3)	2 (13.3)
Familial history of IBD, n (%)	12 (12.6)	4 (13.3)	0 (0.0)
Montreal Classification			
Age at diagnosis			
A1, n (%)	5 (5.3)	-	-
A2, n (%)	71 (74.7)	-	-
A3, n (%)	19 (20.0)	-	-
Disease location		-	
L1, n (%)	26 (27.4)	-	-
L2, n (%)	21 (22.1)	-	-
L3, n (%)	48 (50.5)	-	-
L4, n (%)	4 (4.2)	-	-
Behaviour		-	
B1, n (%)	42 (44.2)	-	-
B2, n (%)	22 (23.2)	-	-
B3, n (%)	29 (30.5)	-	-
Extension			
E1, n (%)	-	3 (10.0)	-
E2, n (%)	-	14 (46.7)	-
E3, n (%)	-	13 (43.3)	-
History of perianal lesions, n (%)	40 (42.1)	-	-
Concomittant therapies			
5-ASA, n (%)	2 (2.1)	7 (23.3)	-
Corticosteroids, n (%)	6 (6.3)	2 (6.6)	-
Budesonide, n (%)	4 (4.2)	0 (0.0)	-
Thiopurines, n (%)	32 (33.7)	8 (26.7)	-
Methotrexate, n (%)	10 (10.5)	0 (0.0)	-
Infliximab, n (%)	40 (42.1)	16 (53.3)	-
Adalimumab, n (%)	16 (16.3)	2 (6.7)	-
Golimumab, n (%)	0 (0.0)	1 (3.3)	-
Ustekinumab, n (%)	1 (1.1)	0 (0.0)	-
Vedolizumab, n (%)	8 (8.4)	4 (13.3)	-
CDAI, median (IQR)	80.0 (24.0–184.0)	-	-
SCCAI, median (IQR)	-	2 (0.5–4.8)	-
Partial Mayo score, median (IQR)	-	1 (0–4)	-
Leucocytes, (Giga/L), median (IQR)	7.28 (5.69–9.55)	6.13 (5.12–7.89)	7.34 (6.30–8.72)
Neutrophils, (Giga/L), median (IQR)	4.41 (3.21–5.75)	3.21 (2.49-4.34)	4.13 (3.49–5.59)
Monocytes, (Giga/L), median (IQR)	0.57 (0.43–0.77)	0.53 (0.42–0.62]	0.50 (0.47–0.68)
Platelets, (cells ×10^3^/L), median (IQR)	292 (242–369)	283 (242–378)	250 (222–270)
CRP, (mg/L), median (IQR)	3.5 (2.9–12.0)	3.0 (1.7–8.1)	2.9 (2.9–2.9)
Faecal Calprotectin, (µg/g) median (IQR)	328 (100–1380)	433 (100–780]	-
Vitamin-D, (µg/L) median (IQR)	20.1 (13.4–27.5)	-	-

SD = standard deviation; IQR = interquartile range; IBD = inflammatory bowel disease; CDAI = Crohn’s disease activity index; SCCAI = simple colitis clinical activity index; CRP = C-reactive protein.

**Table 2:** Prevalence of the CD-associated polymorphisms investigated in the study.

	Crohn’s Disease Patients *n* = 95	Ulcerative Colitis Patients *n* = 30	Healthy Controls *n* = 15	*p*-Value
NOD2				
rs2066844 (snp8)	21 (22.1%)	2 (6.7%)	2 (13.3%)	0.14
rs2066845 (snp12)	8 (8.4%)	3 (10.0%)	0 (0.0%)	0.47
rs2066847 (snp13)	16 (16.8%)	1 (3.3%)	0 (0.0%)	0.044 *
ATG16L1	77 (81.1%)	25 (83.3%)	9 (60.0%)	0.14
IRGM	27 (28.4%)	5 (16.7%)	5 (33.3%)	0.36
ULK1	82 (86.3%)	27 (90.0%)	13 (86.6%)	0.87
LRRK2	7 (7.3%)	2 (6.7%)	1 (6.7%)	0.98
XBP1	13 (13.7%)	5 (16.7%)	1 (6.7%)	0.65
CYLD				
rs17314544	56 (58.9%)	21 (70.0%)	10 (66.7%)	0.51
rs2302759	93 (97.8%)	29 (96.7%)	14 (93.3%)	0.60
rs7205423	66 (69.5%)	22 (73.3%)	12 (80.0%)	0.68
rs12324931	21 (22.1%)	2 (6.7%)	2 (13.3%)	0.14
USP40				
rs12472244	35 (36.8%)	8 (26.7%)	6 (40.0%)	0.54
rs4047198	88 (92.6%)	28 (93.3%)	15 (100.0%)	0.55
rs838548	88 (92.6%)	28 (93.3%)	15 (100.0%)	0.55

* Significant difference between CD patients and both UC patients and healthy controls lumped together; results are given as cumulated prevalence including both heterozygous mutated and homozygous mutated.

**Table 3:** Correlation between bacterial survival and the level of cytokines after 10 h of AIEC LF82 or *E. coli* K-12 infection in MDM from 95 Crohn’s disease patients.

*MDM from CD Patients*		Spearman R	*p*-Value
IL-1β	LF82	0.5398	<0.0001
	K-12	0.08662	0.4449
IL-6	LF82	0.287	0.0061
	K-12	−0.04959	0.6622
IL-10	LF82	0.4638	<0.0001
	K-12	0.02859	0.8025
TNF-α	LF82	0.3142	0.0026
	K-12	0.01768	0.8763

## Supplementary Materials

The following are available online at https://www.mdpi.com/2073-4409/8/11/1394/s1, Table S1: Prevalence of the CD-associated polymorphisms investigated in the study, Figure S1: Western blot and quantification of LC3A/B proteins levels in MDM from CD patients (*n* = 10), UC patients (*n* = 11) and healthy volunteers (*n* = 11), infected during 10 h or not with AIEC LF82 bacteria. The experiment was repeated 3 times.
