# Supplementary material for: Macrophages Inability to Mediate Adherent-Invasive E. coli Replication is Linked to Autophagy in Crohn’s Disease Patients
Source: Cells. 2019 Nov 5;8(11):1394. doi: 10.3390/cells8111394 (PMC6912674; doi:10.3390/cells8111394)
Supplement: Supplementary file 1 [file cells-08-01394-s001.pdf]

Supplementary Material: Figure S1: Western blot and quantification of LC3A/B proteins levels in MDM from CD patients (n=10), UC patients (n=11) and healthy volunteers (n=11), infected during 10h or not with AIEC LF82 bacteria. The experiment was repeated 3 times.

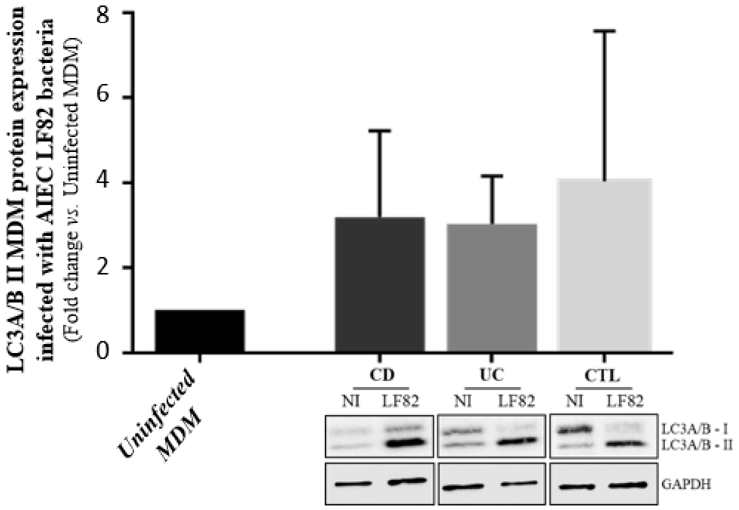

Supplementary Figure S1. Western blot and quantification of LC3A/B proteins levels in MDM from CD patients (n=10), UC patients (n=11) and healthy volunteers (n=11), infected during 10h or not with AIEC LF82 bacteria. The experiment was repeated 3 times.
